# Tetracycline-exposed *Drosophila melanogaster* males produce fewer offspring but a relative excess of sons

**DOI:** 10.1002/ece3.1535

**Published:** 2015-07-14

**Authors:** Kaitlyn L O’Shea, Nadia D Singh

**Affiliations:** Department of Biological Sciences, North Carolina State UniversityRaleigh, North Carolina

**Keywords:** *Drosophila*, fitness, sex ratio, tetracycline, Wolbachia

## Abstract

A large diversity of species possesses endosymbionts; these endosymbionts can exhibit mutualistic, parasitic, and commensal relationships with their hosts. Previous work has consistently revealed that depleting endosymbiont titer with antibiotic treatment can significantly alter host fitness and function, particularly with respect to reproductive phenotypes. Although these findings are often interpreted as resulting from the breakdown of highly coevolved symbioses, it is possible that antibiotic treatment itself rather than endosymbiont removal contributes to the observed perturbations in reproductive phenotypes. Here, we investigate the effect of tetracycline treatment on sex ratio and male reproductive fitness using *Drosophila melanogaster* as a model system. Our results indicate that tetracycline-treated males produce a relative excess of sons. We also find that tetracycline treatment reduces the number of progeny produced by treated males but not treated females. These findings are independent of the effects of tetracycline on Wolbachia titer and implicate the antibiotic itself as mediating these changes. It is yet unclear whether the sex ratio shift and reduction in male reproductive fitness are direct or indirect consequences of tetracycline exposure, and more work is needed to determine the molecular mechanisms by which these disturbances in reproductive phenotypes arise. Our data highlight the importance of considering the potentially confounding effects of antibiotic treatment when investigating the effects of endosymbiont depletion on host phenotypes.

## Introduction

The sex ratio is a critical demographic parameter that is likely to have serious implications for evolutionary dynamics in natural populations. In general, most species tend to show a 1:1 sex ratio as predicted by sex ratio theory (Fisher 1930; Hamilton 1967). However, a variety of mechanisms have been shown to lead to sex ratio distortion in a diversity of organisms. For instance, the sex ratio in many animals has been shown to be sensitive to environmental conditions. With environmental sex determination, the sex ratio is influenced by the environment post-fertilization; temperature has been shown to mediate sex ratio in vertebrates and invertebrates, while other factors such as photoperiod and density frequently mediate sex ratio in invertebrates (for review see Korpelainen 1990). Sequential hermaphroditism is another example of how sex ratios can shift in response to environmental cues and has been observed in a variety of fishes (e.g., Atz 1964). Sex chromosome meiotic drive, the unequal transmission of X- and Y-bearing gametes from the heterogametic sex, is a genetic mechanism by which the sex ratio can be distorted and has been observed in plants and animals alike (for review see Jaenike 2001). Finally, infection with endosymbionts has been shown to alter a variety of host reproductive phenotypes including sex ratio (for review see Engelstadter and Hurst 2009).

Wolbachia is arguably the best-studied endosymbiont with respect to effects on host reproduction. Wolbachia are intracellular bacteria that infect a huge diversity of arthropods and nematodes. Recent evidence suggests that 40–66% of arthropod hosts are infected with Wolbachia (Hilgenboecker et al. 2008; Zug and Hammerstein 2012). These obligate endosymbionts have fascinating effects on the reproductive biology of their host. For instance, Wolbachia has been shown to induce cytoplasmic incompatibility, a form of post-zygotic reproductive isolation that emerges in matings between uninfected females and infected males (e.g., Breeuwer and Werren 1990) or between males and females infected with mutually incompatible strains (Oneill and Karr 1990; Mercot et al. 1995; PerrotMinnot et al. 1996). Wolbachia infections have also been shown to induce parthenogenesis in several haplodiploid taxa including hymenoptera, thrips, and mites (for review see Huigens and Stouthamer 2003; Engelstadter and Hurst 2009), yielding a female-biased sex ratio. Male-killing strains of Wolbachia, which typically lead to male death during embryonic or early larval development, abound in insects, also yield female-biased sex ratios (for review see Kageyama et al. 2012). Female-biased sex ratios can also be induced by Wolbachia-associated feminization, which has been documented in insects and isopods (for review see Kageyama et al. 2012).

The predominantly vertical transmission of Wolbachia (e.g., Richardson et al. 2012) lays the foundation for coevolutionary dynamics between Wolbachia and its hosts. Consistent with the coevolutionary potential latent in symbiotic relationships, studies in whiteflies, silkworm, and Drosophila have indicated that depletion of Wolbachia can alter host reproductive phenotypes including fitness, sex ratio, and mate discrimination (Costa et al. 1997; Puttaraju and Prakash 2005; Miller et al. 2010; Zhong and Li 2013), which would be consistent with a breakdown of coevolutionary dynamics between Wolbachia and its host.

However, depletion of Wolbachia is typically achieved by treatment with the antibiotic tetracycline, and the effects of tetracycline on reproductive phenotypes are largely unexplored. Tetracycline is a broad-spectrum antibiotic that is used to treat a variety of bacterial infections. Tetracycline inhibits action of the prokaryotic 30S ribosome, thus preventing protein synthesis (Brodersen et al. 2000). Exposure to tetracycline has been shown to affect a variety of traits including mitochondrial function (Ballard and Melvin 2007) and larval development (Casiraghi et al. 2002). Although it has recently been shown that tetracycline itself can reduce sperm viability in male pseudoscorpions (Zeh et al. 2012), the effects of tetracycline exposure on the sex ratio have not as yet been explored.

Here, we use *Drosophila melanogaster* as a model system to explore the effects of tetracycline exposure on both the sex ratio and male reproductive fitness. Our experimental design empowers us to separate the effects of Wolbachia depletion from the effects of tetracycline exposure. Our results indicate that tetracycline exposure yields a relative increase in male production and that this effect is driven by tetracycline-exposed males but not females. We further show that male reproductive fitness is compromised following tetracycline treatment. Both of these findings appear unrelated to Wolbachia titer. Although further work is required to determine the mechanism by which tetracycline leads to a male-shifted sex ratio, our findings highlight a novel environmental factor that has the potential to affect the sex ratio. Our results also highlight the importance of considering the confounding effects of tetracycline treatment when investigating the effects of endosymbiont depletion on organismal fitness and function.

## Materials and Methods

### Stocks and fly rearing

The wild-type line used for this experiment was randomly selected from the *Drosophila* Genetic Reference Panel (Mackay et al. 2012). This line is naturally infected with Wolbachia (Huang et al. 2014). Genomic analysis indicates that RAL73 is infected with a *wMel*-like strain of Wolbachia (Richardson et al. 2012). *wMel* has been shown to induce cytoplasmic incompatability, although the magnitude of the effect varies among studies (Bourtzis et al. 1996; Poinsot et al. 1998; McGraw et al. 2002; Reynolds and Hoffmann 2002; Yamada et al. 2007). We created a Wolbachia-free version of RAL73. To clear RAL73 of Wolbachia infection, we raised this strain on tetracycline-containing media for two consecutive generations. These flies were raised in 8-ounce (oz) bottles with a standard cornmeal/molasses media containing tetracycline (dissolved in ethanol) to a final concentration of 0.25 mg/mL media. After the second generation of tetracycline treatment, this stock (denoted RAL73^*w*−^) was raised on standard media for nine generations before the experiment described below began.

### Wolbachia screen

Immediately prior to conducting these experiments, we confirmed the presence of Wolbachia infection in the standard RAL73 line (henceforth denoted RAL73^*w*+^) and the absence of Wolbachia in RAL73^*w*−^ using a PCR-based assay with Wolbachia-specific primers. Four adult females were used per line to test for Wolbachia infection. Briefly, DNA was extracted from each female using a standard squish prep described by G. Gloor and W. Engels (pers. comm.). Each fly was crushed with a motorized pestle and subsequently immersed in a buffered solution (10 mmol/L Tris–Cl pH 8.2, 1 mmol/L EDTA, 25 mmol/L NaCl, 200 *μ*g/mL proteinase K). This was incubated at 37°C for 30 min and then at 95°C for 2 min to inactivate the proteinase K. We used Wolbachia-specific primers wspF and wspR (Jeyaprakash and Hoy 2000) to test for presence/absence of Wolbachia infection.

Amplifying conditions were as follows: 94°/3 min, 12 cycles of 94°/30 sec, 65°/30 sec, 72°/60 sec with the annealing temperature reduced by 1.0 degrees per cycle, followed by 25 cycles of 94°/30 sec, 55°/30 sec, 72°/60 sec. We included a final extension of 72°/7 min. All PCRs were 10 *μ*L, and each contained 5 *μ*L Qiagen 2× PCR Master-Mix, 0.25 *μ*L of each 20 mmol/L primer, 3.5 *μ*L H_2_O, and 1 *μ*L genomic DNA. All four tested RAL73^*w*+^ females showed evidence of Wolbachia infection using this assay, while none of the four tested RAL73^*w*−^ females showed any evidence for Wolbachia infection.

### Experimental crosses

**Figure 1:**
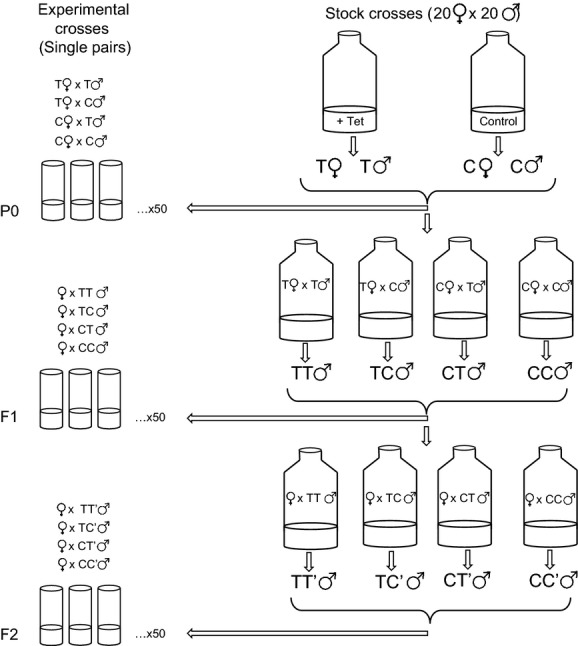
Schematic illustrating the crossing scheme for this experiment. See Methods for complete details.

All crosses were conducted at 25°C on a 12 h/12 h light/dark cycle. A schematic describing the overall experimental design is shown in Fig.1. This experimental regime was conducted in parallel for both RAL73^*w*+^ and RAL73^*w*−^. To generate the P0 flies, virgin females and males were crossed on media with a tetracycline dosage of 0.25 mg tetracycline/mL media. The P0 flies are thus exposed to tetracycline as embryos and larvae. To produce the control P0 flies, virgin females and males were crossed on standard media (with an appropriate amount of ethanol added to control for the vehicle used to administer tetracycline). These crosses were conducted in 8-oz bottles; 20 males and 20 females were used per cross.

Virgin P0 males and females were collected from both the treatment and control bottles and were crossed on standard media in all four possible combinations (control females and control males; control females and treated males; treated females and control males; treated females and treated males). These flies were aged for 24 h, and fifty replicate single-pair matings per mating type were conducted in vials to assay the P0 reproductive output and the sex ratio of progeny produced by the P0 flies. P0 females were allowed to mate and oviposit for 3 days. Reproductive output was estimated as the total number of progeny produced. We estimated sex ratio of those progeny as the male: female ratio.

We independently crossed P0 flies in these four mating combinations in 8-oz bottles to produce F1s (Fig.1). Twenty males and twenty females were used in these crosses. F1 virgin males were collected and aged for 24 h; there are four types of F1 males which we will denote as: (1) CC; (2) CT; (3) TC; and (4) TT, which, respectively, result from crosses between (1) control females and control males; (2) control females and treated males; (3) treated females and control males; and (4) treated females and treated males. These four types of males were each paired with virgin RAL73 stock females in single pairs (50 replicates per cross) in vials to assay the F1 reproductive output and the sex ratio of progeny produced by these four types of F1 males. RAL73^*w*+^ females were used in these crosses when assaying reproductive phenotypes of RAL73^*w*+^ F1 males and RAL73^*w*−^ females were used when assaying reproductive phenotypes of RAL73^*w*−^ F1 males. Females were allowed to mate and oviposit for 3 days.

These four types of males were independently paired with virgin stock females in 8-oz bottles to produce F2 males. RAL73^*w*+^ females were crossed with RAL73^*w*+^ F1 males, and RAL73^*w*−^ females were crossed with RAL73^*w*−^ F2 males. Twenty males and twenty females were used in each of these crosses. Four types of F2 males were produced which we will denote as: (1) CC′; (2) CT′; (3) TC′; and (4) TT′ which, respectively, denote sons of CC, CT, TC, and TT males. Virgin F2 males were collected, aged for 24 h, and subsequently paired with virgin RAL73 stock females in single pairs in vials (50 replicate crosses per cross type) to assay the F2 reproductive output and the sex ratio of progeny produced by these four types of F2 males. RAL73^*w*+^ females were used in these crosses when assaying reproductive phenotypes of RAL73^*w*+^ F2 males, and RAL73^*w*−^ females were used when assaying reproductive phenotypes of RAL73^*w*−^ F2 males. Females were allowed to mate and oviposit for 3 days.

### Statistical analyses

We log-transformed both the total progeny count and the sex ratio for our statistical analyses. To assess the effect of tetracycline treatment on sex ratio and reproductive output in a sex-specific way, we used an analysis of variance (ANOVA). The full model is:

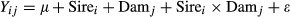
where Sire (*i *=* *1,2) denotes whether or not the male parent in the cross is treated with tetracycline and Dam (*j *=* *1, 2) denotes whether or not the female parent in the cross is treated with tetracycline. Both “Sire” and “Dam” are modeled as fixed effects; the Sire-by-Dam interaction effect is modeled as a fixed effect also. We used this model to separately examine sex ratio and reproductive output as the response variables. We used this model for the P0 data, and also separately for the F1 son and F2 grandson data. Note that when analyzing the F1 sons and F2 grandsons, a significant “sire” effect, for instance, denotes an effect of whether or not the P0 male ancestor was treated with tetracycline.

To separate the effects of tetracycline on reproductive phenotypes from the effects of symbiont depletion, we considered an expanded statistical model. The full model is:

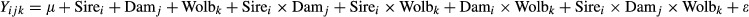
where Sire and Dam are described above and Wolb (*k* = 1, 2) denotes whether the host genotype was infected with Wolbachia at the time at which tetracycline was adminstered. The Sire_*i*_* *× Wolb_*k*_ and Dam_*i*_ × Wolb_*k*_ interaction effects capture whether there are differential effects of tetracycline treatment on Wolbachia-infected versus uninfected males and females, and the Sire_*i*_ × Dam_*j*_ × Wolb_*k*_ factor tests for differential effects of tetracycline treatment in certain combinations of treated/untreated males and females. All of these factors are modeled as fixed effects. We used this model to assess the effects of tetracycline treatment and Wolbachia infection status on both sex ratio and reproductive output. All statistical analyses were conducted in JMP version 10. The data supporting the results of this article are available from the Dryad Digital Repository: http://dx.doi.org/10.5061/dryad.645h2.

## Results and Discussion

Previous work in a variety of organisms indicates that symbiont depletion can alter a variety of reproductive phenotypes including fecundity, sex ratio, and mate discrimination (Costa et al. 1997; Puttaraju and Prakash 2005; Miller et al. 2010; Zhong and Li 2013). These results are often interpreted as resulting from a breakdown of a symbiosis between the focal organism and its likely coevolved endosymbionts. However, one possibility is that at least some of the observed changes in reproductive phenotypes were due to the antibiotic treatment itself rather than the reduction in the endosymbiont titer. Indeed, the antibiotic tetracycline has been shown to have a variety of effects on reproductive phenotypes in both vertebrates and invertebrates (Hargreaves et al. 1998; Farombi et al. 2008; Zeh et al. 2012; Elzeinova et al. 2013). It has further been shown in pseudoscorpions that not only do tetracycline-treated males show reduced sperm viability, but sons of tetracycline-treated males also suffer from reduced sperm viability (Zeh et al. 2012). We tested the extent to which tetracycline treatment alone could alter sex ratio and reproductive output using *D. melanogaster* as a model system and tested the extent to which these changes were preserved across generations. We used a wild-type strain of *D. melanogaster* for these experiments; this strain is naturally infected with Wolbachia. We also cured this line of Wolbachia infection such that we could distinguish between the effects of symbiont depletion and tetracycline exposure on reproductive phenotypes.

### Sex ratio

**Figure 2:**
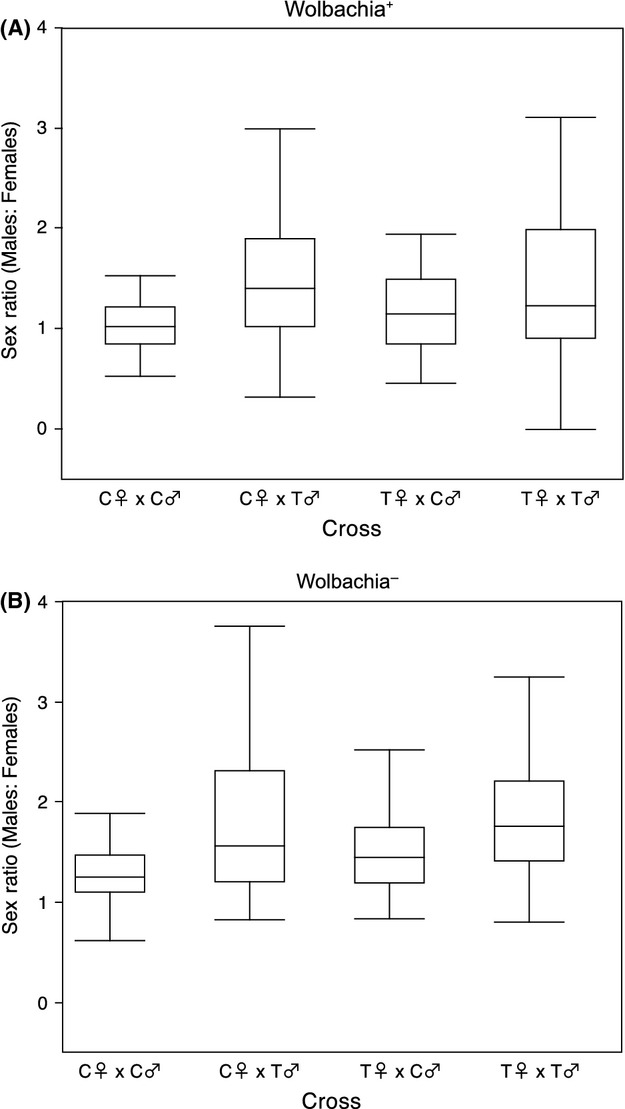
Sex ratio (male: female) of progeny produced by matings of P0 control and treated flies for (A) RAL73^*w*+^ and (B) RAL73^*w*−^. These data are shown in boxplot format, with the median shown as a black line within the box and the edges of the box indicating the 25^th^ and 75^th^ percentile. Whiskers span 1.5 times the interquartile range. Untransformed data are shown.

We exposed RAL73^*w*+^ P0 flies to tetracycline as embryos and larvae (see Methods, Fig.1). We crossed control and tetracycline-treated males and females in all four possible combinations and measured the sex ratio of the progeny produced by these different mating combinations. We estimated sex ratio as the male: female ratio in broods produced by these four types of matings. Interestingly, crosses involving tetracycline-treated fathers show a significant shift toward a relative increase in male production (“sire” effect, *P *=* *0.004, ANOVA) (Fig.2). We find no support for a “dam” effect or a sire-by-dam interaction effect. This bias toward increased relative production of males following tetracycline treatment is consistent with previous results, which consistently show an excess of males produced following tetracycline-induced symbiont depletion (Costa et al. 1997; Puttaraju and Prakash 2005; Miller et al. 2010; Zhong and Li 2013). Although we find the effect driven by sires, previous work shows support for a dam effect (Puttaraju and Prakash 2005; Zhong and Li 2013), which was interpreted as resulting from Wolbachia-mediated cytoplasmic incompatibility.

The observed shift in the sex ratio of offspring produced by tetracycline-treated males could be driven by the presumed reduction in Wolbachia titer resulting from tetracycline treatment or due to the antibiotic treatment itself. To distinguish between these possibilities, we repeated this experiment with RAL73^*w*−^. This strain has the same genetic background as RAL73^*w*+^ but was cured of Wolbachia infection nine generations before the start of the experiment. If the shift in sex ratio is due to the removal of Wolbachia, then tetracycline treatment of RAL73^*w*−^ should not yield a significant shift in the sex ratio of progeny produced by tetracycline-treated P0 flies. However, parallel to our results with RAL73^*w*+^, we find a significant shift toward a relative increase in male production in treated RAL73^*w*−^ P0 flies (Fig.2). This effect is driven by sires (*P *=* *0.0003, ANOVA), and there is no significant effect of dam or sire-by-dam interactions on the sex ratio. These results are robust to method of analysis; a logistic ANOVA on the proportion of males scaled by total progeny count (Littell et al. 2002) confirmed our findings for both RAL73^*w*+^ and RAL73^*w*−^ (data not shown). Our results thus indicate that tetracycline treatment itself can distort the sex ratio toward males in *D. melanogaster*. This distortion is driven by the effects of tetracycline specifically on males. Previous work indicates progeny produced by tetracycline-treated *D. bifasciata* females show no perturbations in sex ratio (Hurst et al. 2000), which is consistent with our finding that the effects of tetracycline exposure on the sex ratio are mediated through sires only.

Although these parallel results in RAL73^*w*+^ and RAL73^*w*−^ P0 flies are suggestive that the shift in sex ratio is independent of Wolbachia depletion, we used an analysis of variance to statistically separate the effects of tetracycline treatment from the effects of endosymbiont removal. We specifically tested whether RAL73^*w*+^ and RAL73^*w*−^ P0 flies showed different effects of tetracycline treatment on the sex ratio (see Materials and Methods), which would be indicative of a role of Wolbachia depletion in altering the sex ratio. Our results indicate that whether or not the host is infected with Wolbachia at the time of tetracycline treatment does not significantly contribute to the observed variation in sex ratio among P0 crosses. There is a strong sire effect (*P *<* *0.0001, ANOVA), but no significant effect of Wolbachia infection status (*P *=* *0.56, ANOVA) or any interactions between infection status and any other model terms (*P *>* *0.36, all interaction effects, ANOVA). Thus, the effects of tetracycline treatment on the sex ratio produced by RAL73^*w*+^ and RAL73^*w*−^ P0 flies are statistically indistinguishable, indicating that Wolbachia infection status at the time of treatment is not contributing to variation in sex ratio. These data thus confirm that tetracycline treatment of males yields a shift in the sex ratio toward a relative increase in male production and that this shift is due to tetracycline treatment itself rather than endosymbiont depletion. More work is needed to determine the molecular mechanisms mediating this phenomenon.

Our finding that tetracycline treatment itself can alter sex ratios suggests that previous results may merit re-investigation. Specifically, in those studies in which males and females were exposed to tetracyline and subsequently assessed for reproductive phenotypes, the effects of tetracycline treatment and the effects of symbiont depletion are impossible to distinguish without further controls (e.g., Costa et al. 1997; Miller et al. 2010; Zhong and Li 2013). This is not to say that endosymbiont depletion did not contribute to the previously observed shift in sex ratio toward males, but rather that the effects of tetracycline exposure may have gone unappreciated because of experimental design. In contrast, studies in which only females are exposed to tetracycline or in which an exposure control is also included (e.g., Hurst et al. 2000) are not expected to be adversely affected by the effects of tetracycline on male reproductive phenotypes that we reveal here.

We also measured the sex ratio of progeny produced by F1 sons and F2 grandsons of tetracycline-treated RAL73^*w*+^ and RAL73^*w*−^ P0 flies. We find no statistical evidence in support of a relative excess of males in the progeny produced by F1 sons (Figure S1) or F2 grandsons (Figure S2) of tetracycline-treated flies relative to descendants of control flies. This indicates that the effects of tetracycline treatment on this particular reproductive phenotype do not persist across generations, at least in this strain of *D. melanogaster*. This finding suggests that studies in which tetracycline is administered and reproductive phenotypes are assessed following several generations of rearing in the absence of tetracycline (e.g., Puttaraju and Prakash 2005) are unlikely to be confounded by any direct effects of tetracycline exposure. Other phenotypes may not prove so robust to this approach; however, mitochondrial metabolism and mitochondrial DNA density in *D. simulans* are significantly perturbed two generations after tetracycline treatment (Ballard and Melvin 2007).

### Reproductive output

**Figure 3:**
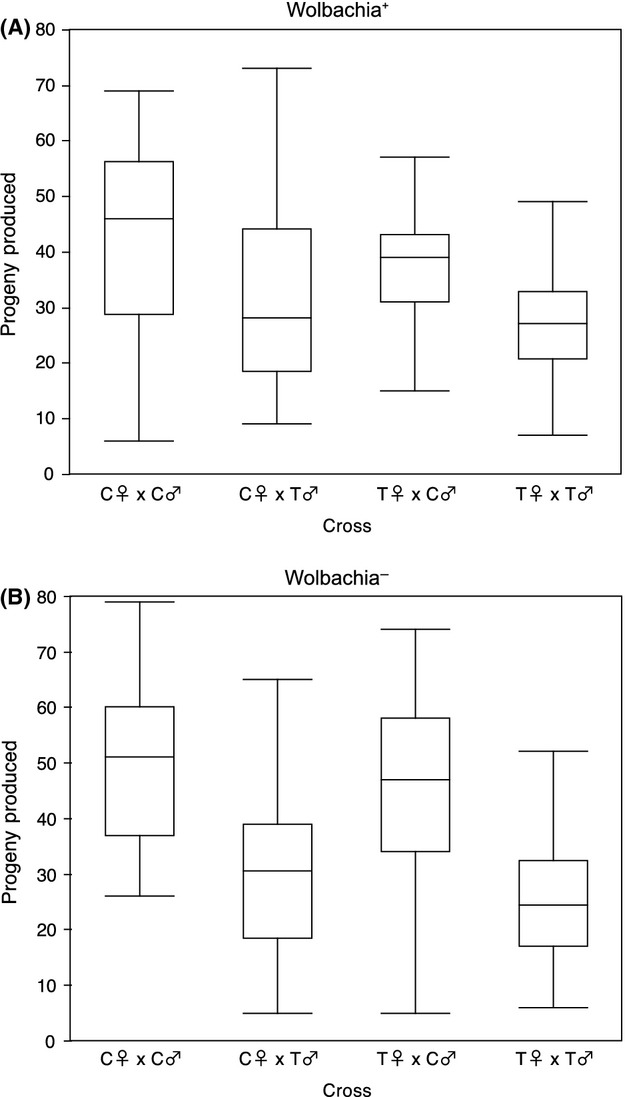
Total number of progeny produced by matings of P0 control and treated flies for (A) RAL73^*w*+^ and (B) RAL73^*w*−^. These data are shown in boxplot format, with the median shown as a black line within the box and the edges of the box indicating the 25^th^ and 75^th^ percentile. Whiskers span 1.5 times the interquartile range. Untransformed data are shown.

As described above, we exposed RAL73^*w*+^ P0 flies to tetracycline and crossed control and tetracycline-treated males and females in all four possible combinations. We measured the reproductive output of the progeny produced by these different mating types. We find a significant “sire” effect (*P *<* *0.0001, ANOVA), with fewer progeny produced in crosses involving a tetracycline-treated male than in crosses involving control males (Fig.3). There is no significant “dam” effect nor a sire-by-dam interaction effect, suggesting that reproductive output of P0 flies is not influenced by whether or not the female had been exposed to tetracycline. Repeating this experiment with RAL73^*w*−^ confirmed the strong effect of tetracycline treatment on male fitness. Although there is no significant “dam” effect, we find a highly significant “sire” effect (*P *<* *0.0001, ANOVA), with fewer progeny produced in crosses involving a tetracycline-treated male than in crosses involving control males (Fig.3). These results are robust to method of analysis; negative binomial regression on transformed or untransformed data yields indistinguishable results from those presented here for both RAL73^*w*+^ and RAL73^*w*−^ (data not shown). Importantly, tetracycline exposure is associated with significant reductions in both the number of males and the number of females for both RAL73^*w*+^ and RAL73^*w*−^ (*P *<* *0.0008, ANOVA, all tests). Moreover, the magnitude of the effect is similar in both sexes (Figure S3), indicating that the tetracycline exposure-associated shift in sex ratio toward a relative increase of males is not, for instance, simply due to the loss of female progeny.

As before, we consider an expanded statistical model to separate the effects of tetracycline treatment from the effects of Wolbachia removal. Specifically, we ask whether RAL73^*w*+^ and RAL73^*w*−^ show differential effects of tetracycline on reproductive output, which would support a role for Wolbachia depletion in reducing male reproductive fitness following antibiotic exposure. We recover a strong sire effect (*P *<* *0.0001, ANOVA) as expected. However, there is no statistical support for a role of infection status on the variation in progeny produced by tetracycline-treated P0 flies (*P *=* *0.48, ANOVA). We do find evidence of a sire-by-Wolbachia interaction effect (*P *=* *0.01, ANOVA), indicating that the reproductive output of males is mediated in part by an interaction between treatment status of the males and Wolbachia infection status at the start of the experiment. Interestingly, these data indicate that Wolbachia-infected control P0 males produce significantly fewer progeny than Wolbachia-cleared control P0 flies (*P *=* *0.02, 2-tailed *t*-test), while there is no significant difference between the number of progeny produced by tetracycline-treated Wolbachia-infected versus Wolbachia-cleared flies (*P *=* *0.20, 2-tailed *t*-test). These data are suggestive of a cost of Wolbachia infection in untreated males with respect to reproductive fitness and also indicate that the reduction in male fitness associated with tetracycline treatment is greater for RAL73^*w*−^ than for RAL73^*w*+^.

It is important to consider our findings in light of cytoplasmic incompatibility that could result from crossing uninfected females with Wolbachia-infected males. *wMel* has been previously shown to be capable of inducing cytoplasmic incompatibility (CI), although the degree of CI observed differs dramatically among studies (Bourtzis et al. 1996; Poinsot et al. 1998; McGraw et al. 2002; Reynolds and Hoffmann 2002; Yamada et al. 2007). However, our results appear robust to any potential effects of cytoplasmic incompatibility. First, as CI is expected to manifest only in crosses of Wolbachia-free females and Wolbachia-infected males, we expect that crosses involving Wolbachia-free females will produce fewer progeny when those females are mated to infected males versus when they are mated to uninfected males. In contrast to this expectation, there is no significant difference in the number of progeny produced between these two types of crosses (*P *=* *0.59, Mann–Whitney *U*-test; Fig.3). Second, that we see statistically equivalent male fitness costs in RAL73^*w*+^ and RAL73^*w*−^ indicates that the observed reduction in fitness associated with tetracycline exposure is not associated with Wolbachia infection and thus is not driven by cytoplasmic incompatibility.

Our data thus indicate that independent of Wolbachia infection, tetracycline treatment strongly perturbs male fitness but does not appear to reduce female fitness as estimated by reproductive output. It is possible that other Drosophila female fitness traits such as fecundity are more severely altered by tetracycline treatment and/or symbiont depletion, which has been seen previously (Puttaraju and Prakash 2005; Miller et al. 2010). Our results are consistent with previous work in pseudoscorpions which found a strong effect of tetracycline treatment on sperm viability but no effect of tetracycline treatment on females (Zeh et al. 2012). Given the critical role of mitochondria in spermatogenesis and sperm motility, and given that tetracycline has been shown to affect mitochondrial function in *Drosophila* (Ballard and Melvin 2007), it is possible that the reduction in male fitness that we observe is due to reduced sperm viability, reduced sperm count, or other sperm-related defects.

We also measured reproductive output of F1 sons and F2 grandsons of tetracycline-treated RAL73^*w*+^ and RAL73^*w*−^ P0 flies. We find no statistical support for a reduction in fitness in F1 sons or F2 grandsons of treated P0 flies (Figures S4, S5), independent of whether the sons/grandsons were descendants of treated P0 males, treated P0 females, or both. This contrasts somewhat with previous work which showed that sons (but not grandsons) of tetracycline-treated fathers also show reproductive impairments (Zeh et al. 2012). It is not as yet clear why we failed to uncover a transgenerational effect of tetracycline treatment on male fitness. If the transgenerational effect of tetracycline treatment on male fitness in pseudoscorpions is mediated by DNA methylation as hypothesized (Zeh et al. 2012), then perhaps no such effect is observed in *D. melanogaster* because of the extremely low levels of DNA methylation (Capuano et al. 2014). It has also been shown that effects of tetracycline can persist longer in some strains of *D. melanogaster* than others (Rottschaefer and Lazzaro 2012). Thus, although we observe no transgenerational effect of tetracycline treatment on male fitness in RAL73, it is certainly possible that other strains could show tetracycline-associated disruptions in male fitness for multiple generations.

Our results thus clearly indicate that tetracycline treatment leads to both a decrease in the number of progeny produced by P0 males and a relative excess of males in those progeny independent of the effects of tetracycline exposure on Wolbachia titer. However, it is not clear whether the effects of tetracycline exposure on progeny count and sex ratio are direct or indirect. It is formally possible that the relative excess of sons produced by tetracycline-treated males is in fact directly due to the decrease in larval density, for instance, rather than being a direct consequence of tetracycline treatment. Although there is no evidence in support of a general relationship between larval density and sex ratio in *D. melanogaster* (Santos et al. 1994), it is possible that such a relationship emerges under certain environmental conditions or in certain genetic backgrounds. Consistent with a role of larval density in mediating sex ratio in the current experiment, we find that the M:F ratio is significantly negatively correlated with progeny count in crosses involving tetracycline-exposed P0 males for RAL73^*w*−^ (Spearman’s *r *=* *−0.38, *P *=* *0.008 for treated males mated to control females; Spearman’s *r *=* *−0.21, *P *=* *0.04 for treated males mated to treated females). However, we fail to recover such correlations in these crosses in RAL73^*w*+^ and also fail to find such correlations in any other cross type. This makes it difficult to assess the relationship between larval density and sex ratio and the implications of that potential relationship for the current study. Thus, although we have demonstrated that tetracycline exposure affects male fitness and the sex ratio of progeny they produce, more work is needed in the future to determine whether these perturbations in reproductive phenotypes are independent effects. Moreover, it will be interesting to determine when the distortion in sex ratio manifests. If tetracycline-exposed males produce an excess of Y-bearing sperm, this would suggest that X-bearing sperm are somehow more susceptible to the effects of tetracycline than Y-bearing sperm. Sexing embryos and larvae at several stages of development will also provide critical information regarding potential viability differences between male and female progeny of tetracycline-exposed males, which could help reveal the mechanisms underlying the tetracycline-associated sex ratio shift we observe here.

## Supporting Information

**Figure S1.** Sex ratio (male:female) of progeny produced by F1 males for (A) RAL73^*w*+^ and (B) RAL73^*w*−^. **Figure S2.** Sex ratio (male:female) of progeny produced by F2 males for (A) RAL73^*w*+^ and (B) RAL73^*w*−^. **Figure S3.** Number of male and female progeny by matings of P0 control and treated flies for (A) RAL73^*w*+^ and (b) RAL73^*w*−^. **Figure S4.** Total number of progeny produced by F1 males for (A) RAL73^*w*+^ and (B) RAL73^*w*−^. **Figure S5.** Total number of progeny produced by F2 males for (A) RAL73^*w*+^ and (B) RAL73^*w*−^.
