# Supplementary material for: Tetracycline-exposed Drosophila melanogaster males produce fewer offspring but a relative excess of sons
Source: Ecol Evol. 2015 Jul 14;5(15):3130–9. doi: 10.1002/ece3.1535 (PMC4559055; doi:10.1002/ece3.1535)

Supplementary Figure 1: Sex ratio (male:female) of progeny produced by F1 males for a) RAL73^{w+} and b) RAL73^{w-}. These data are shown in boxplot format, with the median shown as a black line within the box and the edges of the box indicating the 25th and 75th percentile. Whiskers span 1.5 times the interquartile range. Untransformed data are shown.

a)

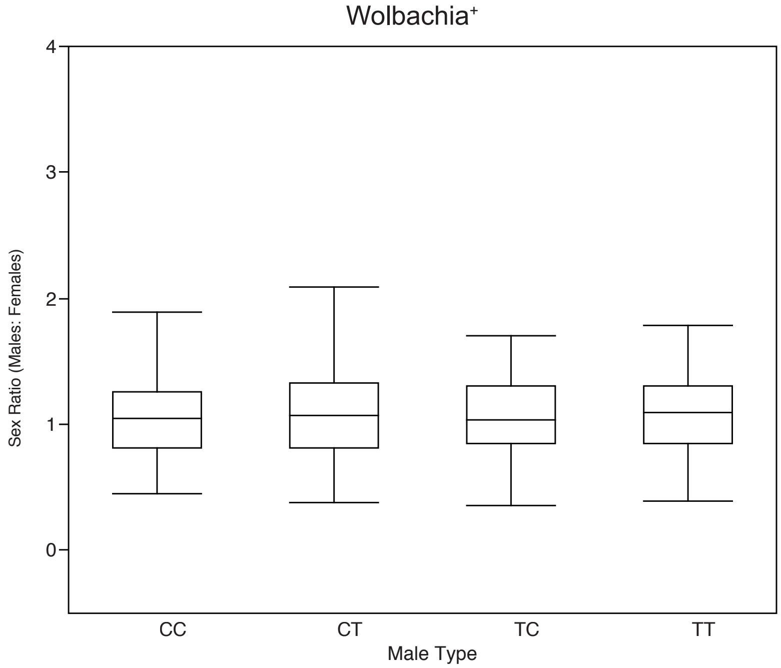

b)

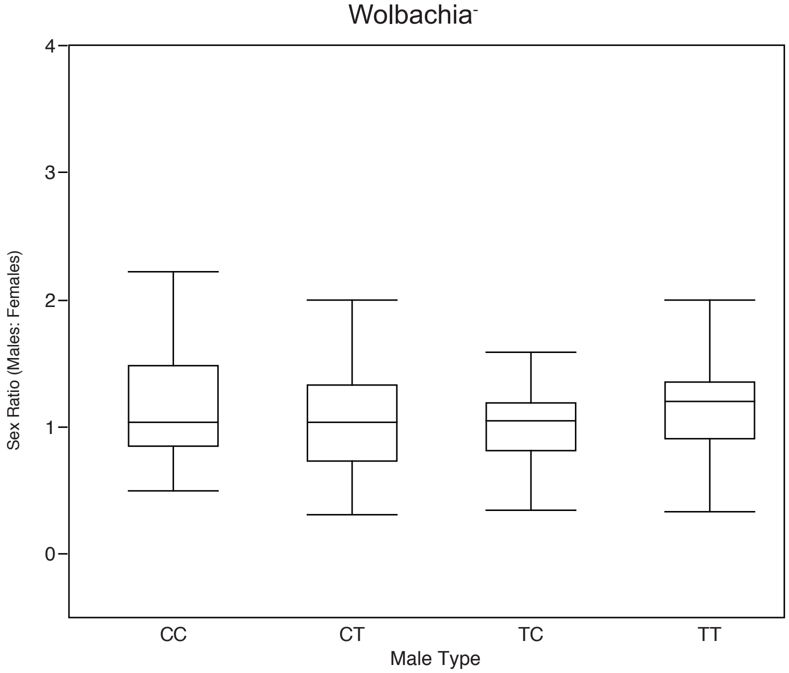

Supplementary Figure 2: Sex ratio (male:female) of progeny produced by F2 males for a) RAL73^{w+} and b) RAL73^{w-}. These data are shown in boxplot format, with the median shown as a black line within the box and the edges of the box indicating the 25th and 75th percentile. Whiskers span 1.5 times the interquartile range. Untransformed data are shown.

a)

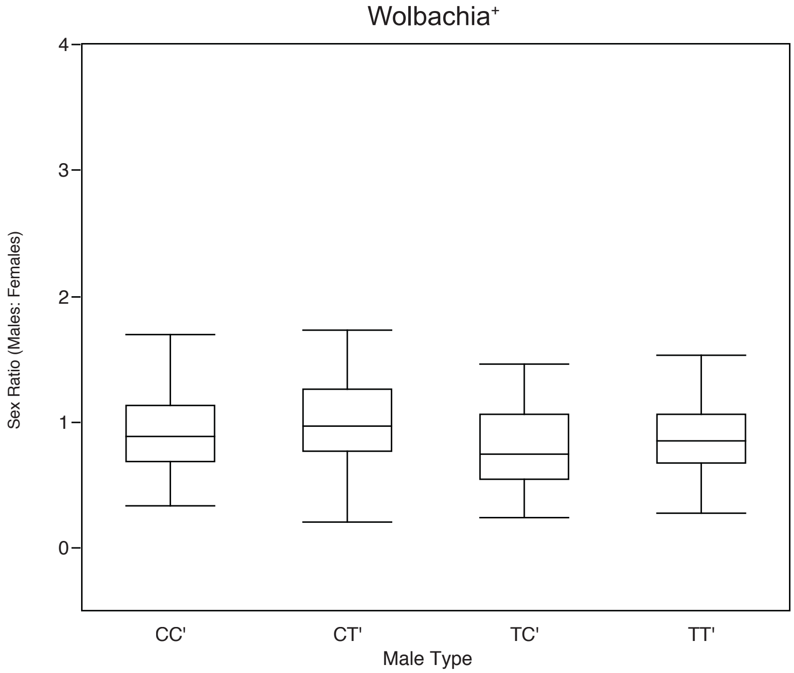

b)

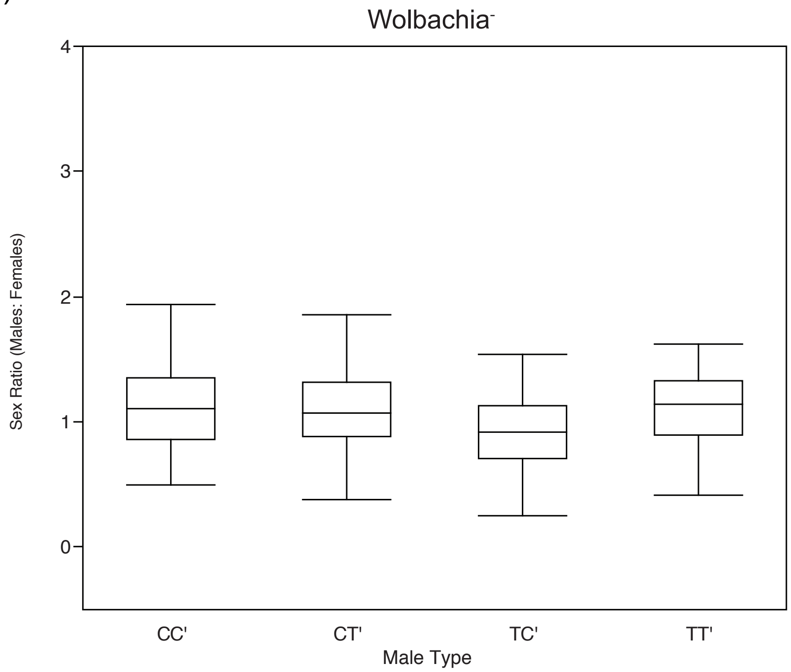

Supplementary Figure 3: Number of male and female progeny by matings of P0 control and treated flies for a) RAL73^{w+} and b) RAL73^{w-}. These data are shown in boxplot format, with the median shown as a black line within the box and the edges of the box indicating the 25th and 75th percentile. The whiskers extend to the most extreme datapoint no farther from the box than 1 times the interquartile range. Untransformed data are shown.

a)

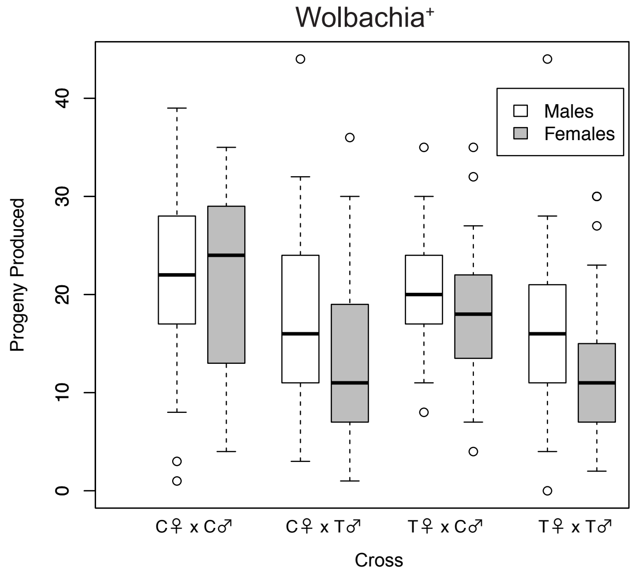

b)

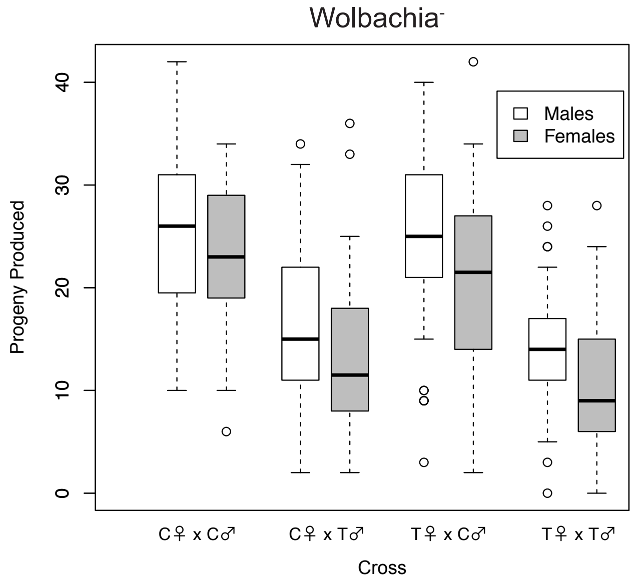

Supplementary Figure 4: Total number of progeny produced by F1 males for a) RAL73^{w+} and b) RAL73^{w-}. These data are shown in boxplot format, with the median shown as a black line within the box and the edges of the box indicating the 25th and 75th percentile. The whiskers span 1.5 times the interquartile range. Untransformed data are shown.

a)

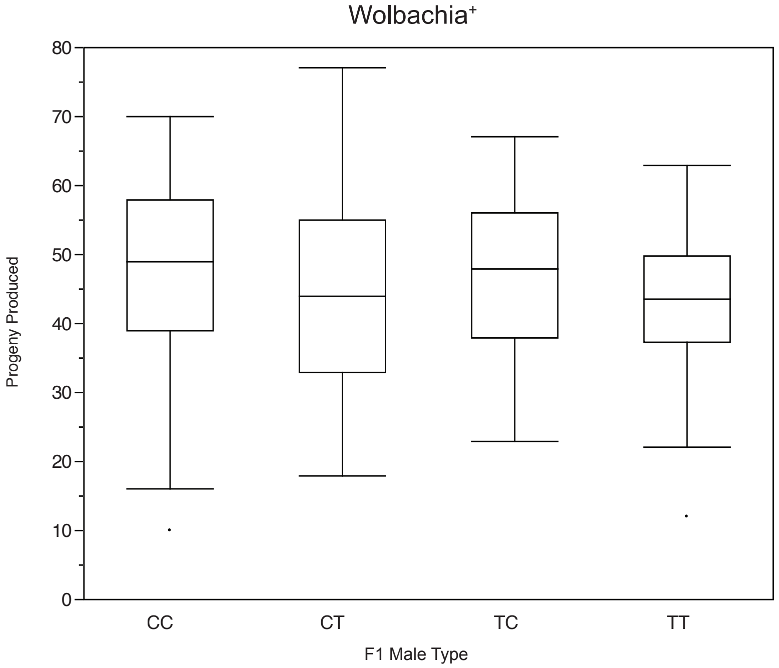

b)

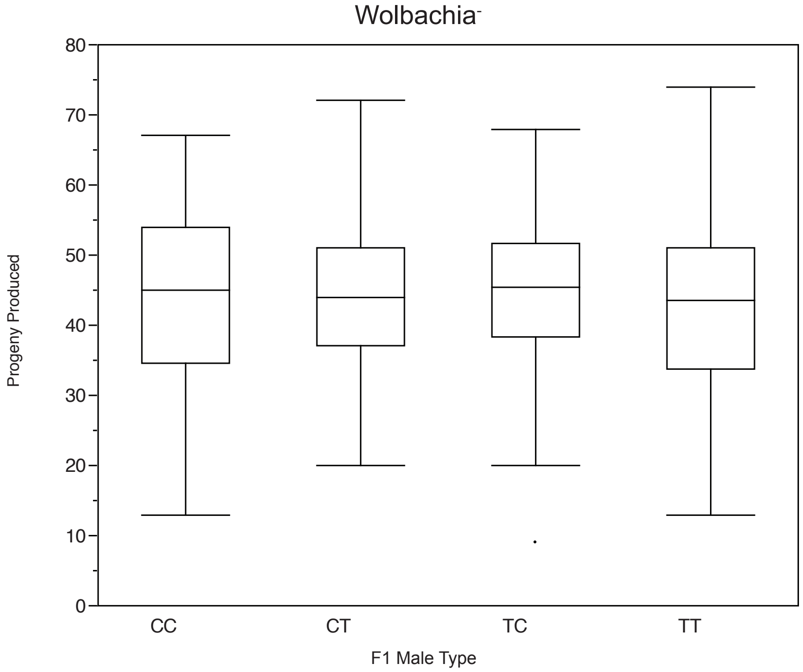

Supplementary Figure 5: Total number of progeny produced by F2 males for a) RAL73^{w+} and b) RAL73^{w-}. These data are shown in boxplot format, with the median shown as a black line within the box and the edges of the box indicating the 25th and 75th percentile. Whiskers span 1.5 times the interquartile range. Untransformed data are shown.

a)

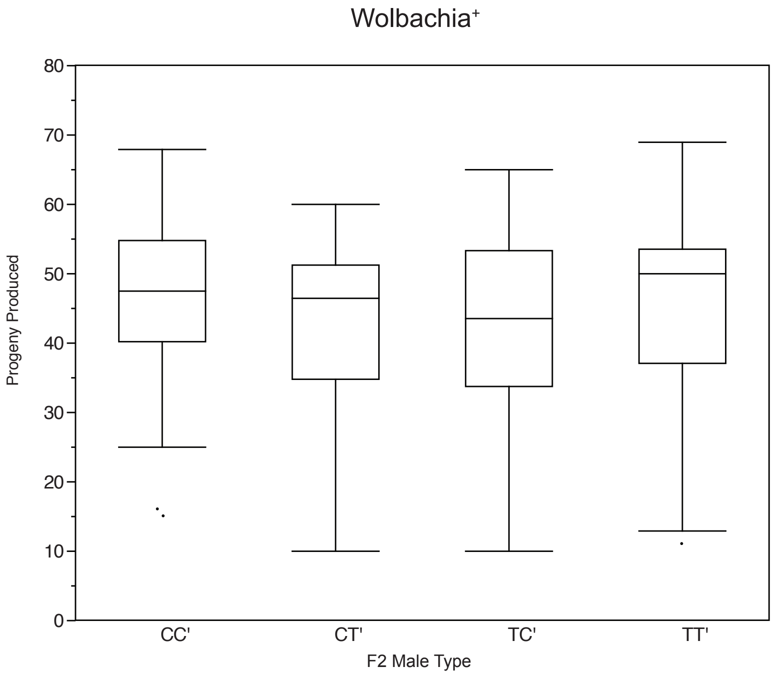

b)

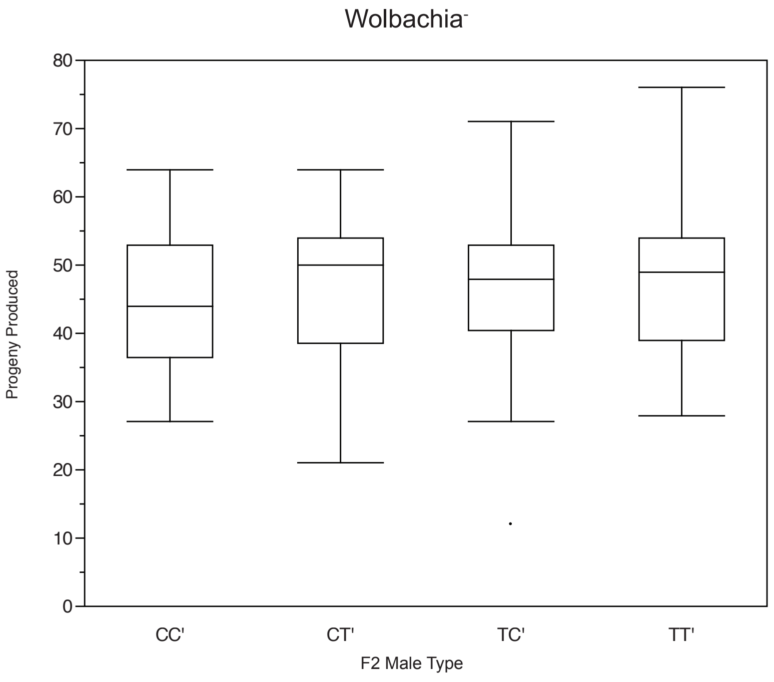

Supplement: Supplementary file 1 [file ece30005-3130-sd1.pdf]
